# Epidemiological and clinical study of cases of endemic pemphigus foliaceus and pemphigus vulgaris in a reference center in the state of Minas Gerais, Brazil^☆^

**DOI:** 10.1016/j.abd.2023.03.004

**Published:** 2023-10-05

**Authors:** Vanessa Martins Barcelos, Everton Carlos Siviero do Vale, Marcelo Grossi Araujo, Flávia Vasques Bittencourt

**Affiliations:** a Service of Dermatology, Hospital das Clínicas, Universidade Federal de Minas Gerais, Belo Horizonte, MG, Brazil; b Postgraduate Programa in Science Applied to Adult Health, Faculdade de Medicina, Universidade Federal de Minas Gerais, Belo Horizonte, MG, Brazil; c Department of Internal Medicine, Faculdade de Medicina, Universidade Federal de Minas Gerais, Belo Horizonte, MG, Brazil

**Keywords:** Autoimmunity, Endemic diseases, Pemphigus, Skin diseases, vesiculobullous

## Abstract

**Background:**

Pemphigus constitutes a group of autoimmune bullous diseases. A reduction in the incidence of endemic pemphigus foliaceus and an increase in pemphigus vulgaris has been described, but there are no studies in Minas Gerais that address the subject.

**Objective:**

To describe the epidemiological and clinical profile of patients with pemphigus treated at the Dermatology Service of a public University Hospital in the state of Minas Gerais, Brazil.

**Methods:**

An observational, descriptive, and cross-sectional study was carried out of cases of endemic pemphigus foliaceus and pemphigus vulgaris, for a period of six months. A questionnaire was filled out with epidemiological and clinical data on the disease.

**Results:**

A total of 122 patients were included in the study, 64 with endemic pemphigus foliaceus and 58 with pemphigus vulgaris. When comparing patients with endemic pemphigus foliaceus and those with pemphigus vulgaris, a statistical difference was observed between the median age of initial disease manifestation (p = 0.001), patient occupation (p = 0.010), area of residence (p = 0.000), forests (p = 0.000) and rivers/streams close to the dwelling (p = 0.001) and the number of systemic medications required to control the disease (p = 0.002). When comparing patients with endemic pemphigus foliaceus to those evaluated in a study carried out at the same service in 2008, there was a statistical difference in the area of residence (p = 0.030).

**Study limitations:**

The assessed population comes from a tertiary care service that is not a reference for the entire state.

**Conclusions:**

Patients with endemic pemphigus foliaceus and pemphigus vulgaris maintain statistically significant differences regarding their main variables in the literature, such as age and area of residence. Historically, there has been a reduction in cases of endemic pemphigus foliaceus and an increase in cases of pemphigus vulgaris in this population.

## Introduction

Pemphigus constitutes a group of autoimmune bullous diseases, characterized by the production of IgG antibodies against desmosomal proteins, especially desmogleins (Dsg). Two main forms are described: pemphigus vulgaris (PV) and pemphigus foliaceus, with the latter showing two epidemiologically distinct forms – classic pemphigus foliaceus and endemic pemphigus foliaceus (EPF).^1^ Patients with PV may have antibodies against Dsg3, characterizing mucosal PV, or against Dsg3 and Dsg1, in the mucocutaneous form. This disease presents with flaccid blisters and erosions in the mucous membranes, or skin and mucous membranes, characterized by suprabasal acantholysis on histopathology. EPF, the main form of pemphigus foliaceus in Brazil, involves the formation of antibodies against Dsg1 and bullous lesions occur exclusively on the skin; subcorneal acantholysis is seen on histopathology.^2^

PV is the most common form of pemphigus worldwide, while pemphigus foliaceus is endemic in Brazil and is described as the main disease variant in the country.^3^ However, there is currently a national reversal trend of incidence between EPF and PV and the term endemic PV has been used in the literature, due to some recent epidemiological similarities between these two diseases, perceived in studies carried out in Brasília (midwestern region) and Ribeirão Preto (southeastern region of the country).^2^, ^4^, ^5^, ^6^

There are no studies in Minas Gerais (southeastern region) that prove this reversal trend, nor any studies that address PV in this state – there are only epidemiological data on EPF. This situation hinders the comparison between these diseases and the understanding of the current epidemiological situation in the region. Data collected in a survey carried out by the Brazilian Society of Dermatology (SBD, *Sociedade Brasileira de Dermatologia*), between July 2013 and June 2014, showed 160 patients with autoimmune bullous diseases at the Dermatology Service of Hospital das Clínicas (HC) of *Universidade Federal de Minas Gerais* (UFMG )/*Empresa Brasileira de Serviços Hospitalares* (EBSERH), 136 of them with pemphigus (85%); 51 with PV (37.5%) and 85 with EPF (62.5%).^7^ These numbers may indicate a higher proportion of EPF compared to PV in years prior to this study.

One of the main purposes of this study was to start a database on patients with PV, aiming to provide comparative references for future analysis of pemphigus in the state. HC-UFMG/EBSERH is a reference service in Minas Gerais and can contribute to the study of PV and EPF. Therefore, the epidemiological and clinical profile of the patients the intention was analyzed to evaluate the characteristics of the two forms of pemphigus, the origin of the attended patients, and the current prevalence of each pemphigus in this sample.

## Methods

An observational, descriptive, and cross-sectional study was carried out to analyze the cases attended at the Dermatology Service of HC-UFMG/EBSERH with a diagnosis of EPF or PV, for a period of six months between April and October 2021. This duration was defined based on the routine follow-up of patients, which occurs with a maximum six-month interval, aiming at including all cases followed at the Service. Patients with a clinical diagnosis of EPF or PV and with histopathological evidence of the disease, who were followed at the Autoimmune Bullous Diseases Outpatient Clinic and at the General Outpatient Clinic of the Dermatology Service of HC-UFMG/EBSERH, between April and October 2021, were invited to participate in the study, regardless of sex and age. Direct immunofluorescence was performed in a minority of patients, due to its irregular availability in the service and, therefore, it was not considered in patient selection. The term EPF was used to characterize all patients with pemphigus foliaceus in this population since the state of Minas Gerais is historically endemic for the disease. Patients who refused to participate in the study were excluded. The research project was approved by the UFMG Research Ethics Committee, under number 4,633,416.

The researcher filled out a questionnaire with information provided by the study participants, which included epidemiological and clinical data about their disease. Data from the medical records were analyzed to complement the information. The obtained variables were included in a database developed using the Excel® software program.

The following variables were evaluated: diagnosis, sex, age, triggering factors, family history of pemphigus, presence of autoimmune disease in 1^st^-degree relatives, level of schooling, profession, area of residence (urban, periurban or rural), forests and rivers or streams close to the dwelling, geographical area where the patient lived when the disease began, initial topography of the lesions, initial clinical stage of the disease (applied only to EPF), number of systemic medications required to achieve disease remission and whether or not the disease recurred during follow-up at the Outpatient Clinic.

The data obtained in relation to EPF were compared to the study carried out at the Service in 2008, regarding the variables: sex, age, family history of pemphigus, occupation and area of residence (urban or peri-urban/rural).^8^

The analysis of categorical variables was performed using frequency and percentage. There were no numerical variables with normal distribution and, for those that did not follow a normal distribution, median values and first and third quartiles were used. The Shapiro-Wilk test was applied to verify the hypothesis of the normality of the variables. The Mann-Whitney test was used to compare EPF and PV in relation to quantitative variables, whereas the chi-square test was used to compare qualitative variables. The significance level in both cases was set at 5%.

Disease onset locations was arbitrarily classified into five regions, based on the grouping of geographically associated mesoregions, aiming to make statistical processing possible, since some of them had very small samples. Therefore, the following regions were defined: Metropolitan Belo Horizonte, Zona da Mata, Vale do Rio Doce, Northern region of Minas Gerais (covering the mesoregions Northwest of Minas, North of Minas, Midwest region, Vale do Mucuri and Jequitinhonha) and Southern region of Minas Gerais (covering the South/Southwest of Minas, Campo das Vertentes, West of Minas and Triângulo/Alto Paranaíba).

Occupations were also aggregated according to the sectors of the economy to which they belonged, based on the National Classification of Economic Activities – IBGE.^9^ In this study, they were grouped into the primary sector (extractive activities, agriculture, and livestock), secondary sector (industry and civil construction), and tertiary sector (services and commerce), in addition to the groups of houseworkers and students.

## Results

Of the total of 124 patients diagnosed with EPF or PV, two refused and 122 accepted the invitation to participate in the study. Of these, 64 were diagnosed with EPF and 58 with PV. As for the refusals, both patients had EPF. The diagnosis was confirmed by histopathological analysis in all cases.

### Endemic pemphigus foliaceus

**Figure 1:**
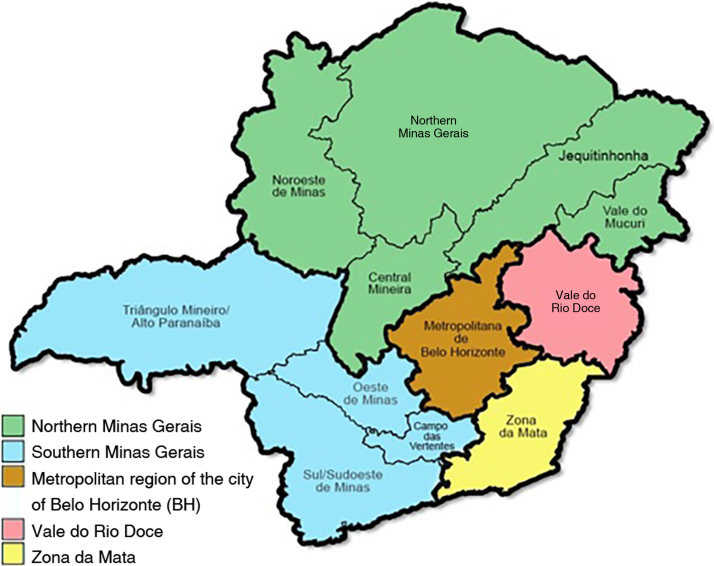
Mesoregions of the state of Minas Gerais and, highlighted in green and blue, the regions arbitrarily demarcated in this study (prepared based on the geographic basis of the Brazilian Institute of Geography and Statistics (IBGE, *Instituto Brasileiro de Geografia e Estatistica*).

Among patients with EPF, 53.1% were female and the median age at disease onset was 30 years (Q1 16.3 – Q3 47.8). The most commonly mentioned triggering factor for disease onset was emotional stress, in 31.3% of patients, followed by sun exposure by 20.3%, and insect bites, in 7.8%. There was a family history of pemphigus in 14.1% of patients and autoimmune disease in a 1^st^-degree relative, in 32.8%. The predominant level of schooling was elementary school (53.1%), and a minority having higher education – only 4.7% of the respondents. As for the higher occupation, workers in the tertiary sector predominated (31.3%), followed by the primary sector (20.3%). Students accounted for 15.6% of the total number of assessed patients with EPF. Most patients reported having lived in rural and peri-urban areas (53.1%), while 46.9% had the urban area as their only place of residence. Accordingly, 71.9% reported forests close to the residence and 54.7% confirmed that there were rivers or streams in the vicinity of the dwelling. Disease onset in most cases occurred in municipalities in the metropolitan region of Belo Horizonte (59.4%), followed by the northern region of the state (14.1%; Fig. 1).

Generalized topography at onset was the most common, occurring in 64.1% of the patients. Following this trend, the classification according to the clinical stage at the time of diagnosis was mostly (76.6%) the generalized bullous-desquamative form, followed by the localized form in 9.4% of the patients. The median number of systemic medications required to achieve disease remission was two and recurrence was observed in 59.4% of cases during patient follow-up.

### Pemphigus vulgaris

Of the 58 patients diagnosed with PV, 46 had the mucocutaneous form and 12 had the exclusive mucosal form, which corresponded to 79.3% and 20.7%, respectively. In this group, 65.5% were female and the median age at disease onset was 45 years. The triggering factors most frequently associated by patients with the onset of the condition were emotional stress (31.0%), sun exposure (17.2%), and physical stress (5.2%). A family history of pemphigus was reported by 8.6% of patients, while a history of autoimmune disease in a 1^st^-degree relative was reported by 29.3%. The predominant level of schooling was elementary school for 58.6% of patients with PV, while higher education was only 3.4%. The predominant occupation in the group was in the tertiary sector (58.6%), followed by an equal percentage of patients in the secondary sector and houseworkes (13.8% each). The vast majority of patients had lived only in the urban area (87.9%) and denied living close to forests (60.3%) or to rivers/streams (74.1%). Most patients (70.7%) reported one of the cities in the metropolitan region of Belo Horizonte as the living quarter at disease onset, while the second most mentioned region was southern Minas, with 10.3% of patients. The most reported initially affected topography was the mucosa, by 75.9% of the interviewees, the oral mucosa in 95.45% of the cases. As for the median number of systemic medications used in the management of patients with PV, it was 2.5 (Q1 2 and Q3 3). The percentage of patients in this group who showed recurrence during follow-up was 79.3%.

### Comparison between endemic pemphigus foliaceus and pemphigus vulgaris

The variables sex, triggering factors, family history of pemphigus or autoimmune diseases, level of schooling, and region of the state where they lived at the time of disease onset did not show any statistically significant differences (Table 1, Table 2).

**Table 1:** Comparison between patients with endemic pemphigus foliaceus and pemphigus vulgaris regarding epidemiological and clinical data, in the Department of Dermatology of HC-UFMG/EBSERH, in 2021

	Diagnosis		
	EPF	PV	p-value
**Sex**			
Male	46.9%	34.5%	0.165
Female	53.1%	65.5%	
**Sunlight exposure**			
No	79.7%	82.8%	0.665
Yes	20.3%	17.2%	
**Physical stress**			
No	95.3%	94.8%	0.902
Yes	4.7%	5.2%	
**Emotional stress**			
No	68.7%	69.0%	0.980
Yes	31.3%	31.0%	
**Medication**			
No	95.3%	96.6%	0.730
Yes	4.7%	3.4%	
**Insect bites**			
No	92.2%	96.6%	0.301
Yes	7.8%	3.4%	
**Family history of pemphigus**			
No	85.9%	91.4%	0.346
Yes	14.1%	8.6%	
**Familial autoimmune disease**			
No	67.2%	70.7%	0.677
Yes	32.8%	29.3%	
**Level of schooling**			
Incomplete Elementary School	45.3%	43.1%	
Complete Elementary School	7.8%	15.5%	
Incomplete High School	12.5%	10.3%	0.846
Complete High School	29.7%	27.6%	
Incomplete Higher Education	3.1%	1.7%	
Complete Higher Education	1.6%	1.7%	
Residence area*			
Urban	46.9%	87.9%	
Rural	32.8%	6.9%	0.000
Periurban	20.3%	5.2%	
Forests*			
No	28.1%	60.3%	0.000
Yes	71.9%	39.7%	
Rivers/streams*			
No	45.3%	74.1%	0.001
Yes	54.7%	25.9%	
Reccurrence*			
No	40.6%	20.7%	0.018
Yes	59.4%	79.3%	

EPF, endemic pemphigus foliaceus; PV, pemphigus vulgaris.

* There is statistical difference between the evaluated groups.

**Table 2:** Comparison between patients with endemic pemphigus foliaceus and pemphigus vulgaris regarding the regions of the state of Minas Gerais where they lived at the disease onset, in the Dermatology Service of HC-UFMG/EBSERH, in 2021

	Diagnosis				
	EPF (n = 64)		PV (n = 58)		
	n	%	n	%	
**Metropolitan region**	38	59.4	41	70.7	
**Northern MG**	9	14.1	4	6.9	χ2 (4.15;4)
**Zona da Mata**	7	10.8	3	5.2	p-value = 0.386
**Vale do Rio Doce**	6	9.4	4	6.9	
**Southern MG**	4	6.3	6	10.3	

EPF, endemic pemphigus foliaceus; PV, pemphigus vulgaris; MG, Minas Gerais.

As for the area of residence reported by the patients, most of the group with EPF lived in rural and peri-urban areas, while the group with PV lived mostly in urban areas, with a p-value = 0.000 (Table 1). Regarding forests and rivers or streams close to the dwelling, there was also a difference between the two groups (p-value = 0.000 and p = 0.001, respectively) – in most patients with EPF, these findings were present, whereas these findings were absent in most patients with PV (Table 1).

There was a difference between the groups of patients with EPF and PV regarding occupation when grouped into primary, secondary, and tertiary sectors (Table 3). Thus, patients with EPF showed a higher concentration in the primary sector, whereas patients with PV prevailed in the tertiary sector. Moreover, 15.6% of the patients with EPF were students, while in PV this proportion was only 1.7%.

**Table 3:** Comparison between patients with endemic pemphigus foliaceus and pemphigus vulgaris regarding occupations and economic sectors, in the Dermatology Service of HC-UFMG/EBSERH, in 2021

	EPF (n = 64)		PV (n = 58)		
	n	%	n	%	
**Houseworkers**	11	17.2	8	13.8	
**Primary sector**	13	20.3	7	12.1	χ2 (13.226;4)
**Secondary sector**	10	15.6	8	13.8	p-value = 0.010
**Tertiary sector**	20	31.3	34	58.6	
**Students**	10	15.6	1	1.7	

EPF, endemic pemphigus foliaceus; PV, pemphigus vulgaris.

The median age of disease onset showed a significant difference between the groups, it was lower in the EPF group (Table 4). Another variable with statistical difference between these two diagnoses was the median number of systemic medications required for disease remission, which was higher in the group with PV. Disease duration (interval between the onset of clinical manifestations and data collection) was longer in the PV group, but there was no significant difference between the groups.

**Table 4:** Comparison between patients with endemic pemphigus foliaceus and pemphigus vulgaris regarding age at disease onset, disease duration and number of systemic medications required for disease remission, in the Dermatology Service of HC-UFMG/EBSERH, in 2021

	Median (Q1 – Q3)		
	EPF	PV	p-value
**Age (years)**	30 (16.3 – 47.8)	45 (33 – 55.3)	0.001
**Disease duration (years)**	4 (4 – 14.8)	8 (5 – 13.3)	0.949
**Systemic medications**	2 (2 – 2)	2.5 (2 – 3)	0.002

EPF, endemic pemphigus foliaceus; PV, pemphigus vulgaris.

The recurrence rate also differed between the groups, it was more common in PV than in EPF patients (p-value = 0.018; Table 1).

### Comparison with the reference study

The epidemiological study carried out at the same Service as a Master's degree dissertation in 2008 included only patients with EPF. Therefore, the present study used as a comparative sample only the 64 participants with the same diagnosis. The reference study was designed in two stages, the first studied the sample from the Dermatology Outpatient Clinic of HC-UFMG/EBSERH (94 participants) while in the second, data from other services in the state were evaluated (161 participants).^8^ The comparison with the present study was performed only with patients from the first stage.

The results showed that there was no difference regarding sex (p = 0.889) and age distribution at disease onset (p = 0.241) between the compared groups (Table 5).

**Table 5:** Comparison between patients with endemic pemphigus foliaceus in the reference study and the current study regarding the epidemiological characteristics, in the Dermatology Service of HC-UFMG/EBSERH

	Pimentel (2008)	Current study (2021)	
**Sex**			
Female	51 (54.3%)	34 (53.1%)	χ2 (0.0196;1)
Male	43 (45.7%)	30 (46.9%)	p-value = 0.889
**Age (years)**			
0 to 12	7 (7.4%)	4 (6.3%)	
13 to 21	18 (19.1%)	18 (28.1%)	χ2 (6.74;5)
22 to 45	44 (46.8%)	25 (39.1%)	p-value = 0.241
46 to 65	15 (15.9%)	13 (20.3%)	
>65	4 (4.4%)	4 (6.3%)	
Unknown	6 (6.4%)	0	
**Family history of pemphigus**			
Yes	7 (7.4%)	9 (14.1%)	
No	78 (83%)	55 (85.9%)	-
Unknown	9 (9.6%)	0	
**Occupation**			
Houseworkers	23 (24.5%)	11 (17.2%)	
Primary sector	19 (20.2%)	13 (20.3%)	-
Secondary sector	4 (4.3%)	10 (15.6%)	
Tertiary sector	27 (28.7%)	20 (31.3%)	
Students	11 (11.7%)	10 (15.6%)	
Unknown	10 (10.6%)	0	
Residence area*			
Rural/Periurban	40 (42.6%)	34 (53.1%)	χ2 (7.04;2)
Urban	45 (47.9%)	30 (46.9%)	p-value = 0.030
Unknown	9 (9.5%)	0	

* There is statistical difference between the evaluated groups.

The only comparatively analyzed variable between the two groups, which showed statistical difference was the area of residence, in which urban areas predominated in the reference group and rural areas in the current one (p = 0.030), regardless of the unknown data in the 2008 study.

A family history of pemphigus was not compared between the groups because 9.6% of the data were not reported in the previous study and the proportion of cases with a positive family history was low in both studies (<15%). As for occupation, the primary and tertiary sectors showed similar percentages; however, percentages were not compared because unknown data in the previous study reached 10.6%.

## Discussion

Recently, in Brazil, the reversal trend in incidence between EPF and PV has been discussed, with reports suggesting that the latter, as the number of cases increases, presents now epidemiological characteristics similar to those of EPF.^6^ A study published in 2007, which evaluated patients at a hospital in Brasília (midwestern region), showed there were individuals with clinical, histopathological, and serological characteristics suggestive of PV, but similar epidemiology to that of EPF, concluding this is a rare form of endemic PV, using this term for the first time in the literature.^4^ Later studies carried out in Ribeirão Preto, São Paulo (southeastern Brazil), found similar results.^5^, ^6^

Historically, Brazilian studies highlight the higher prevalence of EPF in the country in relation to PV, with reports of 17:1 ratios in some rural areas of Brazil; however, it can be observed that the number of patients diagnosed with EPF and PV in the present study, is similar.^3^ This allows the inference that PV prevalence in the institution may be increasing over the years, as the 2008 study did not consider including this disease, due to the lack of a representative sample. In addition, in the data survey carried out for the SBD between 2013 and 2014, the proportion of PV was 37.5% and PF was 62.5%, while in the current study, this proportion is 47.5% and 52.5%, respectively.^7^

In contrast, in the current study, important epidemiological differences were observed between EPF and PV, such as age at disease onset, area of residence (urban × periurban/rural), and presence of forests, rivers or streams close to the living quarters, which wouldn’t allow the term “endemic” to characterize PV patients at this institution. On the other hand, the present study was able to show that endemic is adequate to characterize PF patients in the study.

### Epidemiological characteristics

The population in this study had a higher frequency of females in both types of pemphigus, lower in EPF and higher in PV, but without a statistically significant difference. In the 2008 study at the Service, a slight predominance of female individuals with EPF was also observed, but without a statistical difference, when comparing the number of individuals in the state of Minas Gerais by sex.^8^ The literature does not describe differences regarding sex in EPF, unlike PV, in which females.^10^, ^11^, ^12^, ^13^

The age at EPF onset, in this sample, is significantly younger than that of PV, in agreement with the literature data. Studies show EPF occurring in younger individuals, with a peak incidence in the 2^nd^ and 3^rd^ decades of life, while PV peaks around the 4^th^ to 6^th^ decades.^11^, ^13^, ^14^, ^15^ Despite that, recent Brazilian studies suggest a decrease in the age range of PV involvement in some regions of the country, such as a study carried out in the southeastern region of the state of São Paulo, which showed 17.7% of patients presenting PV before the age of 30.^6^, ^16^

More studies are needed to understand the triggering and aggravating factors for pemphigus, as recognizing them would allow identifying which situations could lead to a breakdown of immune tolerance and, therefore, to the induction of pemphigus. This would allow better patient guidance.^17^ Emotional stress is widely recognized in the literature and was the factor most often cited by patients in this study; it can be explained by the increase in endogenous corticosteroids in stressful situations, which would lead to an increase in pemphigus-inducing cytokines.^17^, ^18^ Despite this hypothesis, physical stress was identified as a trigger by a minority of patients. Ultraviolet radiation is also another well-established factor, and in this study, sun exposure was the second most reported factor in both groups.^17^ As for drug exposure, several studies place them as the main triggers, but few patients actually admit this association, as shown in this study, which could be explained by memory bias.^13^, ^17^

Many studies on EPF mention the association of hematophagous insect bites, such as the genus *Simulium* (blackflies).^19^ A case-control study found that blackfly bites were 4.7 times more frequent in patients with EPF than in individuals without pemphigus.^20^ Another study found that individuals who have the HLA-DRB1 allele and who are repeatedly bitten by hematophagous insects have a higher relative risk for the production of IgM and IgE against salivary antigens of these insects.^3^ The antibodies produced against antigens LJM11 and LJM17, present in the salivary glands of *Lutzomyia longipalpis* (sandfly, transmitter of leishmaniasis), may interact with Dsg1, due to structural similarities between these proteins, which could trigger the disease by cross-reaction.^21^ Despite that, this factor was rarely mentioned by the EPF patients in this study, with no significant difference when compared to the PV group.

A family history of EPF is often mentioned in the literature, with reports of 18.05% in a study carried out in 1972, in Goiás (midwestern region).^22^ In the 2008 study, carried out at the same institution as this study, familial involvement was observed in 7.45% of patients with EPF, a lower percentage than that found in the present study (14.1%).^8^ As for PV, there are rare reports of familial cases, but in this sample, the percentage was 8.6% and there was no statistical difference in relation to EPF. This increased percentage could be attributed to the small population sample assessed in the study. PV is found to be more prevalent in patients with certain HLA alleles, such as HLA-DRB1*0402 (more prevalent among Ashkenazi jews), HLA-DRB1*1401, HLA-DRB1*1404, and HLA-DQB1*050323.^23^ Despite this fact, the exact correlation between a particular genetic profile and a specific clinical profile has not been elucidated, and other non-HLA genes are also possibly involved, as this disease has been described as polygenic.^17^, ^24^ Studies that have reported familial cases of PV and investigated the related HLA alleles, found no correspondence between them.^4^, ^25^, ^26^, ^27^ There have also been studies that demonstrated an association of EPF with specific HLA alleles, such as HLA-DRB1*0404, HLA-DRB1*1402, and HLA-DRB1*1406, with a relative risk for EPF of 14.^3^, ^28^ The genetic background of the participants was not included in this study.

Pemphigus is an autoimmune bullous disease and, due to this etiopathogenic characteristic, there have been many reports of a family history of autoimmune disease, with thyroid diseases being the most common.^13^, ^17^ In this sample, a considerable percentage of association was observed between EPF and PV with autoimmune disease in the family; approximately 30% of patients in each group reported it.

The predominant level of schooling obtained in both groups of patients was incomplete or complete elementary school, which may reflect the socioeconomic profile of the assessed population, coming from a reference service of the Brazilian Unified Health System (SUS, *Sistema Único de Saúde*). This variable was not addressed by Pimentel (2008); however, it is believed to be important epidemiological data due to the sociodemographic characteristics usually related to patients diagnosed with EPF.

In relation to occupation, most patients worked in the tertiary sector, which is the main economic sector in Brazil. However, there was a predominance of occupation in the primary sector among patients with EPF, in agreement with the literature, which describes this disease as predominant among rural, agricultural and road construction workers.^14^ Moreover, studies have shown that, historically, the disease accompanied extractive activities, with a reduction in the number of cases in urbanized areas.^14^, ^19^ PV, in turn, is a disease more often found in urban areas, which justifies the lower percentage of patients in the primary sector of the economy. As for students, they are more frequently found in the EPF group, which is in line with the fact that the age range for this disease is younger. These differences were statistically significant.

For many years, a tendency for EPF to be concentrated in rural areas has been described, including better disease control when the patient lives in more urbanized regions.^19^ This study corroborates these reports, as it is observed that most patients with EPF lived in peri-urban and rural areas, in contrast to PV patients, who were mostly urban. As expected, the presence of forests, rivers, or streams close to the dwelling is also more often observed in the EPF than in the PV group, as the data also described in the literature.^19^

The restriction of endemic EPF to certain mesoregions and areas of the country has been the subject of several studies, but none has been able to elucidate the etiological factors involved; the cause of the disease and why it occurs in certain regions remains unknown.^8^ There was no difference between the living quarters at disease onset between EPF and PV regarding the five regions determined in this study. However, this variable is influenced by the fact that only patients from a single service in the state were studied, which does not necessarily attends patients from all regions of Minas Gerais.

### Clinical characteristics

In this study, the initial topography of EPF involvement was predominantly the generalized form, consisting of disseminated lesions on the face, trunk, and limbs in the first weeks after the onset of the condition, increasing gradually.^3^, ^14^, ^29^ The literature describes that most patients persist with the localized form of the disease, in disagreement with what was observed in this study, which can perhaps be explained by the fact that the sample is from a tertiary care service that attends more serious demands.^29^ As for the PV group, the oral mucosa was the site of initial involvement in most patients, in agreement with the literature, which describes this anatomical site as the initial topography in 70% of cases.^12^ A total of 20.7% of patients with PV had the exclusive mucosal form, which could be justified by earlier diagnosis of cases that sought care at the service.

PV is recognized as a disease with higher morbidity than EPF and is considered more difficult to control.^30^ This is corroborated in the present study, as the number of different systemic medications required for disease remission was greater in PV and disease recurrence during patient follow-up was also more frequent in the PV group.

### Reference study

The study by Pimentel (2008) was conducted at the Dermatology Service of the HC-UFMG/EBSERH in a context where patients with EPF predominated to the detriment of those with PV.^8^ The study assessed only EPF patients, which limited the comparison to just this diagnosis.

Data showed no significant change regarding sex and age among EPF patients who attended the Service in the last 15 years, considering the period assessed in each study.

However, regarding the area of residence, the reference group showed that most patients were from urban areas (47.9%), while in the current study, most were from peri-urban and rural areas (53.1%; p-value = 0.030). This difference can be explained by the fact that the present study included periurban areas in the rural area group, while the previous study did not make this distinction.

The present study suggests an increase in the number of cases of PV in the Service in recent years and is a pioneer in the state of Minas Gerais. It also demonstrates that the epidemiological and clinical characteristics of PV maintain important differences from those of EPF. Unfortunately, there is no previous documentation of PV to carry out a comparative study between previous and current prevalence rates, but the obtained data will contribute to future research with this objective.

The study population is considered to be representative of the state, as it shows similarities to the sample of the study carried out in the Service in 2008, which inferred that the Dermatology Outpatient Clinic of HC-UFMG/EBSERH attends a representative sample of patients with pemphigus from the state of Minas Gerais.^8^

The limitations inherent to a cross-sectional study and the fact that the assessed population came from a tertiary care hospital, which is not a reference for all regions of the state, are emphasized, and it is recommended that further studies evaluate patients from representative locations throughout the entire state of Minas Gerais, aiming to confirm the observed trend.

There is a memory bias for data collection, in addition to the fact that the grouping of residence locations in urban and peri-urban/rural areas does not consider temporary exposures during leisure or whether there was temporary residence in other regions, which may represent an analysis bias.^8^

Also noteworthy is the fact that the data collection was carried out over a period of six months, in addition to having been conducted during the COVID-19 pandemic, which may have influenced the demand for medical care during the data collection period.

Nonetheless, this study is considered important in the scenario of autoimmune bullous diseases in Brazil, since pemphigus, especially PV, has high morbidity and, in many cases, a diagnostic delay leads to loss of quality of life and increased risk of adverse events caused by medications used for its treatment. It is also emphasized the importance of better understanding the epidemiological and clinical characteristics of these diseases at the state level, aiming to optimize their recognition and management.

## Conclusion

This study evaluated the epidemiological and clinical characteristics of patients with EPF and PV treated at the Dermatology Service of HC-UFMG/EBSERH, pioneering the epidemiological study of PV in the state of Minas Gerais.

Based on the analysis of the results, it is suggested that they are in agreement with the recent national literature, which indicates a reversal trend of prevalence between EPF and PV.

The epidemiological similarities between PV and EPF cases, with some authors suggesting the use of the term ‘endemic PV’, were not identified in this study, in disagreement with recent Brazilian studies – the groups maintain statistically significant differences regarding the main variables, such as age and area of residence. In contrast, the term “endemic” has shown to be adequate to characterize the cases of PF in this study, which showed disease onset at a younger age predominantly living in rural and peri-urban areas, close to forests and watercourses.

Studies that evaluate patients from reference services and specialists representing all regions of Minas Gerais are necessary to obtain knowledge about pemphigus that is closer to the state reality.

## Financial support

None declared.

## Authors' contributions

Vanessa Martins Barcelos: Design and planning of the study; collection, analysis, and interpretation of data; statistical analysis; drafting and editing of the manuscript; critical review of the literature; approval of the final version of the manuscript.

Everton Carlos Siviero do Vale: Design and planning of the study; collection, analysis, and interpretation of data; effective participation in research orientation; intellectual participation in the propaedeutic and/or therapeutic conduct of the studied cases; critical review of the manuscript; approval of the final version of the manuscript.

Marcelo Grossi Araújo: Collection, analysis and interpretation of data; effective participation in research orientation; critical review of the manuscript; approval of the final version of the manuscript.

Flávia Vasques Bittencourt: Effective participation in research orientation; critical review of the manuscript; approval of the final version of the manuscript.

## Conflicts of interest

None declared.

## References

[1] Pires C.A., Viana V.B., Araújo F.C., Müller S.F., Oliveira M.S., Carneiro F.R. (2014). Evaluation of cases of pemphigus vulgaris and pemphigus foliaceus from a reference service in Pará state, Brazil. An Bras Dermatol..

[2] Celere B.S., Vernal S., La Serra L., Franco Brochado M.J., Moschini L.E., Roselino A.M. (2017). Spatial distribution of pemphigus ocurrence over five decades in Southeastern Brasil. Am J Trop Med Hyg..

[3] Hans-Filho G., Aoki V., Bittner N.R.H., Bittner G.C. (2018). Fogo selvagem: endemic pemphigus foliaceus. An Bras Dermatol..

[4] Rocha-Alvarez R., Ortega-Loayza A.G., Friedman H., Campbell I., Aoki V., Rivitti E.A. (2007). Endemic pemphigus vulgaris. Arch Dermatol..

[5] Gonçalves G.A., Brito M.M., Salathiel A.M., Ferraz T.S., Alves D., Roselino A.M. (2011). Incidence of pemphigus vulgaris exceeds that of pemphigus foliaceus in a region where pemphigus foliaceus is endemic: analysis of a 21-year historical series. An Bras Dermatol..

[6] Celere B.S., Vernal S., Brochado M.J.F., Segura-Muñoz S.I., Roselino A.M. (2017). Geographical foci and epidemiological changes of pemphigus vulgaris in four decades in Southeastern Brazil. Int J Dermatol..

[7] Sociedade Brasileira de Dermatologia. Levantamento sobre atendimento de dermatoses bolhosas autoimunes nos serviços credenciados de dermatologia do Brasil. Rio de Janeiro. Comunicação pessoal.

[8] Pimentel L.C.F. (2008).

[9] ftp.ibge [Intenet]. Brasil: Instituto Brasileiro de Geografia e Estatística; c2023. Classificação Nacional de Atividades Econômicas. Versão 2.0. 2ª edição. [Cited 2023 Mar 1]. Available from: https://ftp.ibge.gov.br/Informacoes_Gerais_e_Referencia/Classificacoes/CNAE/cnae2_0_2edicao/cnae2_0_2edicao_20150609.pdf.

[10] Kasperkiewicz M., Ellebrecht C.T., Takahashi H., Yamagami J., Zillikens D., Payne A.S. (2017). Pemphigus. Nat Rev Dis Primers..

[11] Kridin K. (2018). Pemphigus group: overview, epidemiology, mortality, and comorbidities. Immunol Res..

[12] Porro A.M., Hans Filho G., Santi C.G. (2019). Consensus on the treatment of autoimmune bullous dermatoses: pemphigus vulgaris and pemphigus foliaceus – Brazilian Society of Dermatology. An Bras Dermatol..

[13] James K.A., Culton D.A., Diaz L.A. (2011). Diagnosis and clinical features of pemphigus foliaceus. Dermatol Clin..

[14] Diaz L.A., Sampaio S.A., Rivitti E.A., Martins C.R., Cunha P.R., Lombardi C. (1989). Endemic pemphigus foliaceus (fogo selvagem). I. Clinical features and immunopathology. J Am Acad Dermatol..

[15] Pollmann R., Schmidt T., Eming R., Hertl M. (2018). Pemphigus: a comprehensive review on pathogenesis, clinical presentation and novel therapeutic approaches. Clin Rev Allergy Immunol..

[16] Porro A.M., Seque C.A., Ferreira M.C.C., Enokihara M.M.S.E.S. (2019). Pemphigus vulgaris. An Bras Dermatol..

[17] Tavakolpour S. (2018). Pemphigus trigger factors: special focus on pemphigus vulgaris and pemphigus foliaceus. Arch Dermatol Res..

[18] Venugopal S.S., Murrel D.F. (2011). Diagnosis and clinical features of pemphigus vulgaris. Dermatol Clin..

[19] Aoki V., Rivitti E.A., Diaz L.A., Cooperative Group on Fogo Selvagem Research (2015). Update on fogo selvagem, an endemic form of pemphigus foliaceus. J Dermatol..

[20] Lombardi C., Borges P.C., Chaul A., Sampaio S.A., Rivitti E.A., Friedman H. (1992). Environmental risk factors in endemic pemphigus foliaceus (fogo selvagem). “The Cooperative Group on Fogo Selvagem Research”. J Invest Dermatol..

[21] Diaz L.A., Prisayanh P., Qaqish B., Temple B.R., Aoki V., Hans-Filho G. (2020). A Lutzomyia longipalps salivary protein induces cross-reactive antibodies to pemphigus autoantigen desmoglein 1. J Invest Dermatol..

[22] Auad A. (1972). Pênfigo foliáceo Sul-Americano no Estado de Goiás. Rev Pat Trop..

[23] Lee E., Lendas K.A., Chow S., Pirani Y., Gordon D., Dionisio R. (2006). Disease relevant HLA class II alleles isolated by genotypic, haplotypic, and sequence analysis in North American Caucasians with pemphigus vulgaris. Hum Immunol..

[24] Sinha A.A. (2011). The genetics of pemphigus. Dermatol Clin..

[25] Laskarus G., Sklavounou A., Stavrou A., Stavropoulou K. (1989). Familial pemphigus vulgaris with oral manifestations affecting two Greek families. J Oral Pathol Med..

[26] Katzenelson V., David M., Zamir R., Mellibovsky J., Idises C., Sandbank M. (1990). Familial pemphigus vulgaris. Dermatologica..

[27] Starzycki Z., Chorzelski T.P., Jablonska S. (1998). Familial pemphigus vulgaris in mother and daughter. Int J Dermatol..

[28] Moraes M.E., Fernandez-Vina M., Lazaro A., Diaz L.A., Filho G.H., Friedman H. (1997). An epitope in the third hypervariable region of the DRB1 gene is involved in the susceptibility to endemic pemphigus foliaceus (fogo selvagem) in three different Brazilian populations. Tissue Antigens..

[29] Campbell I.T., Reis V., Aoki V., Cunha P.R., Hans Filho G., Alves G.F. (2001). Endemic pemphigus foliaceous / fogo selvagem. An Bras Dermatol..

[30] Murrel D.F., Peña S., Joly P., Marinovic B., Hashimoto T., Diaz L.A. (2020). Diagnosis and management of pemphigus: recommendations of an international panel of experts. J Am Acad Dermatol..

